# Injection Molding of Thermoplastic Cellulose Esters and Their Compatibility with Poly(Lactic Acid) and Polyethylene

**DOI:** 10.3390/ma11122358

**Published:** 2018-11-23

**Authors:** Pia Willberg-Keyriläinen, Hannes Orelma, Jarmo Ropponen

**Affiliations:** VTT Technical Research Centre of Finland Ltd., Tietotie 4E, P.O. Box 1000, FI-02044 VTT, FI-02150 Espoo, Finland; hannes.orelma@vtt.fi (H.O.); jarmo.ropponen@vtt.fi (J.R.)

**Keywords:** cellulose, long chain cellulose esters, thermoplastic, injection molding, mechanical properties

## Abstract

Interest in biobased polymers from renewable resources has grown in recent years due to environmental concerns, but they still have a minimal fraction of the total global market. In this study, the injection molding of thermoplastic cellulose octanate (cellulose C8) and cellulose palmitate (cellulose C16) were studied. The mechanical properties of injection-molded test specimens were analyzed by using tensile testing, and the internal structure of injection-molded objects was studied by using a field emission scanning electron microscopy (FE-SEM). We showed that thermoplastic cellulose C8 and cellulose C16 were completely processable without the addition of a plasticizer, which is very unusual in the case of cellulose esters. The compatibility of cellulose esters with poly(lactic acid) (PLA) and biopolyethylene (bio-PE) was also tested. By compounding the cellulose esters with PLA, the elongation of PLA-based blends could be improved and the density could be reduced. The tested thermoplastic cellulose materials were fully biobased, and have good future potential to be used in injection molding applications.

## 1. Introduction

Biobased polymers are materials that are produced from renewable resources. The interest in biobased polymers has grown in recent years due to environmental concerns, but they still have a minimal fraction of the total global plastic market. However, the market of biobased plastics has recently experienced a period of rapid growth [1]. 

Cellulose is one of the most abundant natural polymers on earth, and hence can be regarded as an important raw material in multiple products such as textiles, papers, foods, cosmetics, and biomaterials [2]. Wood materials, which are the most abundant source for cellulose, have a resistant microfibril network that gives cellulose its natural strength and reactivity [3]. However, pure cellulose is not directly processable in injection molding. One of the unique properties of cellulose is that it is chemically tailored to have a required function, and it also has thermoplasticity [4,5]. Cellulose thermoplastic behavior can be improved, for example, by using long chain esterification [6,7]. Long chain cellulose esters with a chain length of fatty substituents ≥C6 (cellulose hexanoate) are biobased materials originating from renewable materials [8]. Moreover, there is already commercially available shorter chain length cellulose acetate (CA), cellulose acetate propionate (CAP), and cellulose acetate butyrate (CAB), which are thermoplastically processable.

In our previous studies, we developed molar mass-controlled long chain cellulose esters, which were processable without the addition of a plasticizer [6,7]. The processing of cellulose esters without plasticizer is very unusual, and typically plasticizers (e.g., triethyl citrate, polyethylene glycol) are required when CA, CAP, and CAB are processed [9,10,11].

Currently, the most widely used thermoplastic material is polyethylene (PE), mainly due to its competitive price and suitability for several applications [12]. Polyethylene can also be produced from biobased resources such as sugarcane or maize. Bio-PE has the same technical properties as petrochemical polyethylene and can be used in the same applications as fossil-based PE [1,13]. Another well-known thermoplastic polymer, poly(lactic acid) (PLA), is a biopolymer that has been considered to be one of the most promising materials to replace petroleum-based plastics in many applications such as biomedical compounds, packaging, and textile fibers [14,15,16,17]. PLA is a biodegradable material produced from renewable resources such as corn starch or sugarcane, with high tensile strength properties [13]. Its mechanical properties are similar to or better than those of, for example, polypropylene [18]. The disadvantages of PLA are its brittleness, low thermal stability [15,18], and narrow processing window [14,19].

Composite materials are commonly used in several applications, including airplanes and automobiles. Most of the commercial composites are carbon or glass fiber-reinforced [20]. Due to the difficulties of recycling these materials, natural fibers (e.g., cellulosic fibers) from renewable resources are nowadays widely studied as reinforcements in polymeric composites. For example, PLA/cellulosic fiber [14,18,21], high-density polyethylene (HDPE)/cellulosic fiber [12,22,23], low-density polyethylene (LDPE)/cellulosic fiber [24], and PLA/nanocellulose [25,26] have been reported. Cellulosic fiber-reinforced polymeric composites are currently used in applications in the construction and automotive industries [27,28,29]. Cellulose fibers have many advantages such as good mechanical properties, biodegradability, and lightness compared to commonly used glass and inorganic fibers [24,30,31]. Additionally, flexibility of natural fibers during processing does not cause any harm to the processing equipment [27]. However, due to cellulose hydrophilicity and the hydrophobic nature of synthetic polymer, composites usually exhibit poor surface adhesion between fibers and the polymeric matrix, which reduces their potential as reinforcing agents [27,32]. In order to improve fiber and polymeric matrix adhesion, physical or chemical surface modification of natural fibers is often needed [33,34,35]. 

**Figure 1:**
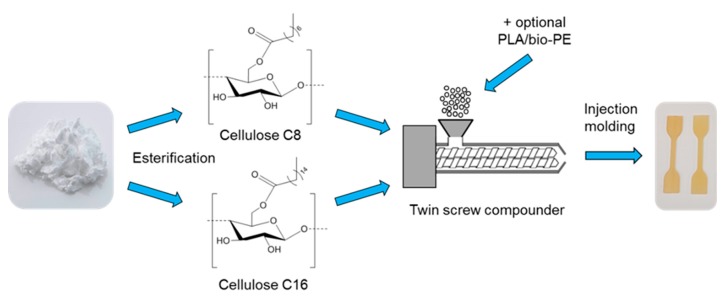
Schematic illustration of this research. PLA: poly(lactic acid); PE: polyethylene.

In this study, the injection molding of thermoplastic cellulose C8, cellulose C16, and their blends were studied (Figure 1). The compounded test specimens were prepared by injection molding, and the mechanical properties (elastic modulus, tensile strength, elongation, and impact strength) were analyzed. The compatibility of cellulose esters with PLA and bio-PE and the effect of cellulose esters on PLA and bio-PE mechanical properties were also evaluated.

## 2. Materials and Methods 

### 2.1. Materials

Commercial softwood dissolving grade pulp produced by Domsjö Fabriker AB, Örnsköldsvik, Sweden, was used for esterification. Prior to esterification, the pulp was ozone-pretreated according to a method described by Willberg-Keyriläinen et al. [6]. All other reagents were analytical grade and purchased from Sigma-Aldrich, Helsinki, Finland, and were used as received. Poly(lactic acid) grade 3052D was provided by Natureworks, USA. Biobased high-density polyethylene grade SHA7260 (the bio-PE) was provided by Braskem, Boa Vista, Brazil.

### 2.2. Methods

#### 2.2.1. Preparation of Cellulose Esters

The homogenous esterification of the cellulose octanate (cellulose C8) and cellulose palmitate (cellulose C16) was conducted in a bench scale using the method presented by Willberg-Keyriläinen et al. [6,7]. Cellulose was first dissolved in 5% LiCl/DMAc solution. Then, anhydrous pyridine (3.6 equivalent to cellulose anhydroglucose unit (AGU)) and octanoyl or palmitoyl chloride (3 equivalent to cellulose AGU) were added to the cellulose solution. The reaction was carried out at 80 °C for 16 h. 

#### 2.2.2. Solid State Nuclear Magnetic Resonance (ssNMR)

The prepared cellulose esters were characterized using the solid state ^13^C cross polarization magic angle spinning (CP/MAS) NMR spectroscopy with an Agilent 600 MHz NMR spectrometer (Agilent Technologies, Palo Alto, CA, USA), using a 3.2 mm magic angle spinning (MAS) probe head. For both cellulose esters, 10,000 scans were accumulated using 10 s recycle time, 3 ms contact time, and a MAS rate of 10 kHz. 

#### 2.2.3. Compounding

Compounds were produced using a twin screw microcompounder (Vari-BatchTM 15, DSM Xplore, Geleen, the Netherlands, batch volume 15 mL), a screw speed of 100 rpm, and a screw residence time of 1 min. Compounding temperature was 130 °C or 180 °C for the different compounds. All materials were dried in a vacuum oven at 40 °C overnight prior to use. Detailed parameters of used blends and temperatures are presented in Table 1. 

#### 2.2.4. Injection Molding

Injection moldings were conducted using a microinjection molding machine (Minijet Type 557-2270, Haake ThermoFisher Scientific, Cheshire, UK). The standard test samples were molded for tensile strength testing and impact strength testing. In injection molding, the mold temperature was 35 °C. An injection pressure of 400 bar for 5 s and a holding pressure of 200 bar for 15 s were used. 

#### 2.2.5. Scanning Electron Microscopy (SEM)

Scanning electron microscopy analysis of the injection molded samples was performed with a field emission SEM (FE-SEM, Merlin^®^, Carl Zeiss GmbH, Jena, Germany) using 2 kV acceleration voltage. Prior to imaging the cross-sections, the samples were freeze-fractured under liquid nitrogen and sputter-coated (SCD 050, Balzers AG, Balzers, Liechtenstein) with a thin gold layer.

#### 2.2.6. Density

Densities of the injection molded samples were measured according to the Archimedean principle by weighing the sample in the air and in ethanol. The temperature of the ethanol was 22 °C and 0.7888 g/cm^3^ was used as the density of ethanol.

#### 2.2.7. Tensile Strength Testing 

Tensile strength tests were performed using an Instron 4505 Universal Testing machine (Instron, Buckinghamshire, UK) according to the ISO 527 standard. A 10 kN load cell and a 5 mm/min crosshead speed were used. Five replicate standard dog bone-shaped samples (gauge dimensions of 25 mm × 4 mm × 2 mm) were tested. The test specimens were kept in standard conditions (23 °C, 50% relative humidity) for one week before testing.

#### 2.2.8. Impact Strength Testing

Impact strength tests were performed using a Charpy Ceast Resil 5.5 impact test machine (CEAST, Pianezza, Italy) according to the ISO 527 standard. Five replicate un-notched standard test sample bars (80 mm × 10 mm × 4 mm) were tested using a 1–5 J hammer. The test specimens were kept in standard conditions (23 °C, 50% relative humidity) for one week before testing.

## 3. Results and Discussion

### 3.1. Preparation of Cellulose Esters

Commercial softwood pulp was ozone-treated to decrease its molar mass. This pretreatment was performed according to the method described in our previous article [6]. The molar mass of the starting cellulose before ozone treatment was 520 kDa, and after ozone treatment was 58 kDa (Figure S1, Supplementary Materials). Cellulose esters were prepared using a homogeneous method according to our earlier study [7], with side chain lengths C8 (octanate) and C16 (palmitate). The degree of substitution (DS) of the samples was analyzed using solid state NMR spectroscopy. The NMR spectra with peak assignment are presented in Supplementary Materials (Figure S2). The main difference between the C8 and C16 spectra could be observed between 20 and 35 ppm. The reason was that the chain length of C16 was two times longer than C8, and CH_2_ shifts of the side chain of cellulose esters occurred in this range. DS values were calculated by comparing the cellulose esters carbonyl carbon (173 ppm) integral to the cellulose C1 signal (105 ppm) integral. According to the NMR results, the DS values of cellulose C8 and cellulose C16 were 1.5 and 1.2, respectively. These DS values were much lower compared to reported commercial thermoplastic celluloses (CAP and CAB) [36,37]. 

### 3.2. Injection Molding of Cellulose Esters

In this research, our target was to study in detail the processability and mechanical properties of cellulose C16 and cellulose C8 esters. In our previous research [7], we reported that these cellulose esters (cellulose C8 and C16) were completely processable without the addition of a plasticizer, which is very unusual in the case of cellulose esters. Both cellulose esters were compounded at 130 °C as pure material and as blends, using 25, 50, and 75 wt % ratios in order to study the full range of composition variations. After compounding, the test specimens were prepared by injection molding.

**Figure 2:**
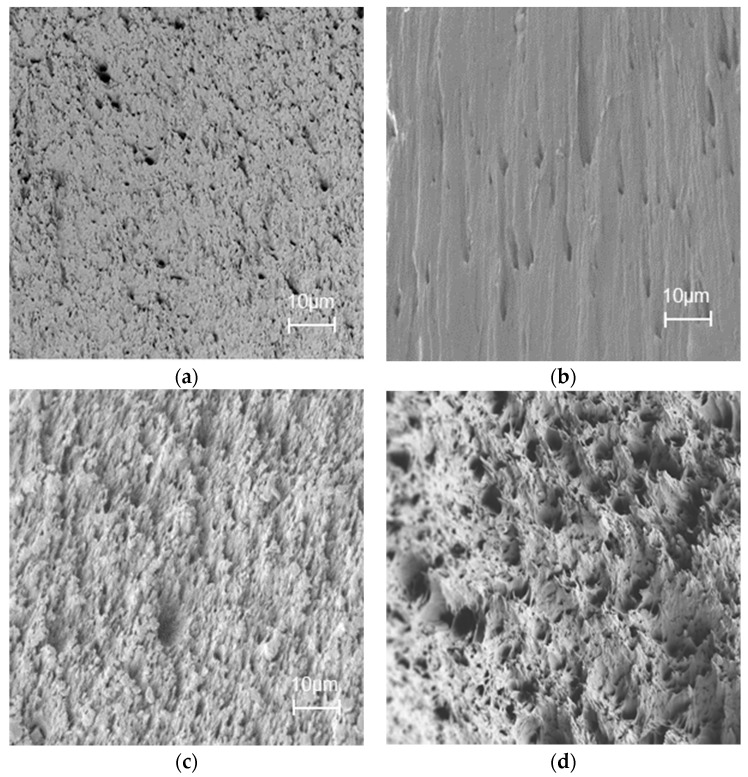

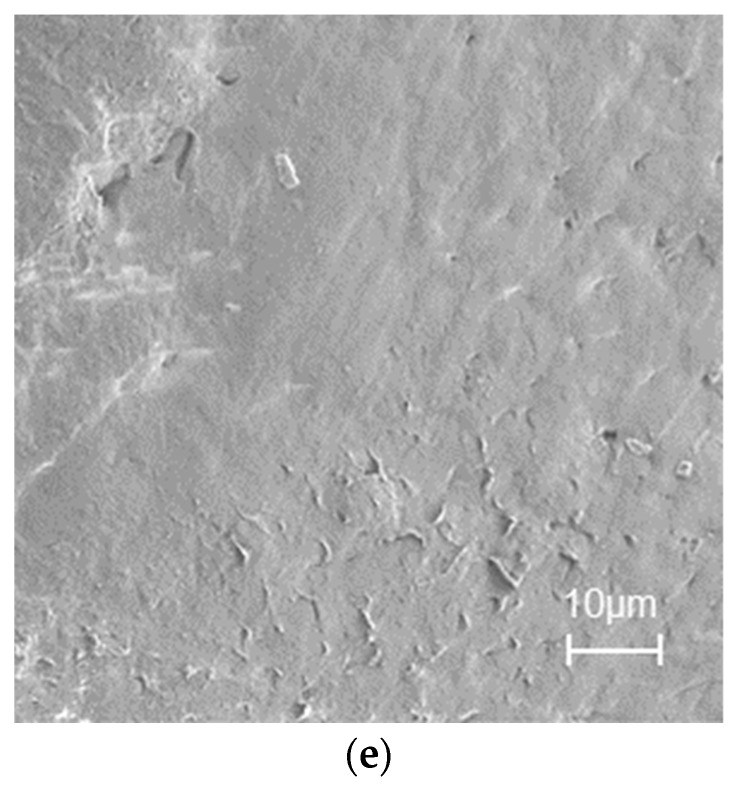
SEM images of injection-molded cellulose ester blends at 1000× magnification (scale bar = 10 µm): (**a**) Cellulose C16; (**b**) cellulose C8; (**c**) cellulose C16/C8, 75%:25%; (**d**) cellulose C16/C8, 50%:50%; (**e**) cellulose C16/C8, 25%:75%.

The morphology of injection-molded cellulose ester samples was studied using scanning electron microscopy. Cross-sectional surface cuts of the injection-molded samples were imaged, and SEM images were taken at 1000× magnification. According to the SEM results, the cross-sections of pure cellulose C16 were more porous than those of cellulose C8 (Figure 2a,b). The images indicated that the prepared cellulose materials were well-compounded and melted, although the compounding time was quite short (1 min). The cellulose C8 and C16 were also compatible together, and no large-scale unevenness was observed in the SEM images (Figure 2c–e). The 75:25 wt % mixing ratio of C16/C8, respectively, decreased the smoothness of the surface. The same trend continued linearly when the C8 content was increased to 50 wt %, and larger pores were observed. However, when the C8 content increased to 75 wt %, the surface was rather smooth, indicating excellent compatibility.

### 3.3. Mechanical Properties of Injection-Molded Cellulose Esters 

**Figure 3:**
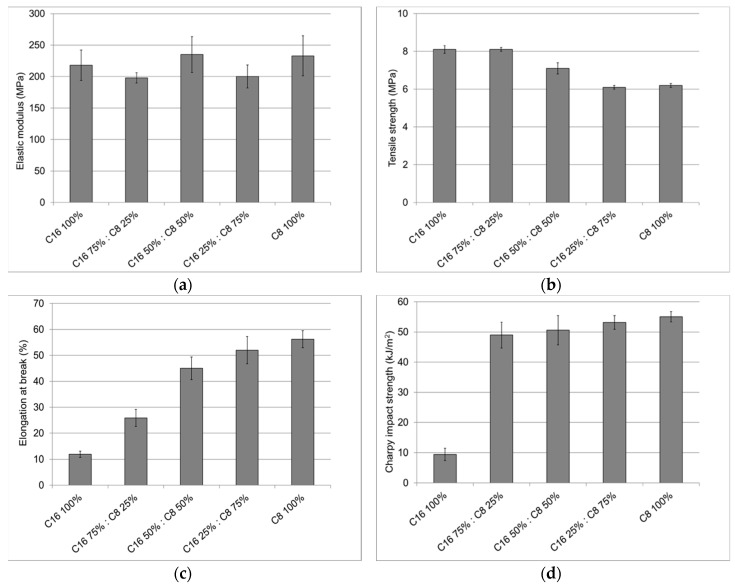
Mechanical properties of injection-molded cellulose C8 and C16 ester samples and their mixed blends: (**a**) Elastic modulus; (**b**) tensile strength; (**c**) elongation at break; (**d**) Charpy impact strength.

The mechanical properties, elastic modulus, tensile strength, elongation at break, and Charpy impact strength of the injection-molded cellulose ester test specimens were determined. The elastic modulus (Figure 3a) of cellulose C16 and cellulose C8 were approximately at the same level (0.2 GPa), and no significant effect of side chain length on elastic modulus was observed. However, in the tensile strengths, side chain length had a small effect (Figure 3b). As for cellulose C16 and C8, the tensile strengths were 8 MPa and 6 MPa, respectively. This observation is in line with our previous research [6], in which we investigated the effect of the side chain length on the mechanical properties of cellulose ester films. However, the earlier reported tensile strength values of cellulose ester films were much higher (>20 MPa) than these results for injection-molded samples. This is probably due to the fact that the applied injection molding process at elevated temperature had an effect on the produced cellulose ester specimens. Degradation temperatures (*T_deg_*) of cellulose C8 and C16 were between 230 and 270 °C [6]. However, it is likely that some degradation occurred at lower temperatures, which could be observed as a color becoming darker. When cellulose C16 and cellulose C8 were compounded together, elastic modulus and tensile strength remained almost constant, which indicated good compatibility of these esters.

The major difference between cellulose C8 and cellulose C16 was observed in the elongation values (Figure 3c). Cellulose C16 had five times lower elongation than cellulose C8. We made the same observation in our previous research [7]. Furthermore, the elongations of the specimens increased linearly with an increasing amount of cellulose C8 in the cellulose C16/C8 blends.

The elongation values of the cellulose C8 and C16 esters were much higher than reported for commercially available shorter chain-length CA, CAP, and CAB [38,39,40], but the elastic modulus and tensile strength values were lower. This is in line with an earlier reported observation [41] stating that when elongation values increased, elastic modulus and tensile strength decreased. 

The impact strength of pure cellulose C8 was higher than that of cellulose C16 (Figure 3d). However, even a small amount of cellulose C8 had an effect on the impact strength of C8/C16 blends. A 25 wt % cellulose octane addition to the cellulose C16 already increased the impact strength to the same level as that of pure cellulose C8.

### 3.4. The Compatibility of Cellulose Esters with PLA and Bio-PE 

The compatibility of cellulose C16 and cellulose C8 with PLA and bio-PE was also tested. PLA and PE are among the most studied biobased polymer matrices. To the best of our knowledge, long chain cellulose esters in the blends with other polymer matrices have not been published earlier. However, shorter cellulose alkyl esters have been studied in blends, for example with polycaprolactone (PCL) [42,43], with PLA [44,45], and with HDPE [46].

Cellulose C16 and cellulose C8 were compounded with PLA and bio-PE using 25, 50, and 75 wt % ratios. Cellulose esters were compounded at 130 °C, but a higher temperature was required to further compound these cellulose esters with PLA and bio-PE, and therefore 180 °C was used. After compounding, the test specimens were prepared by injection molding.

**Figure 4:**
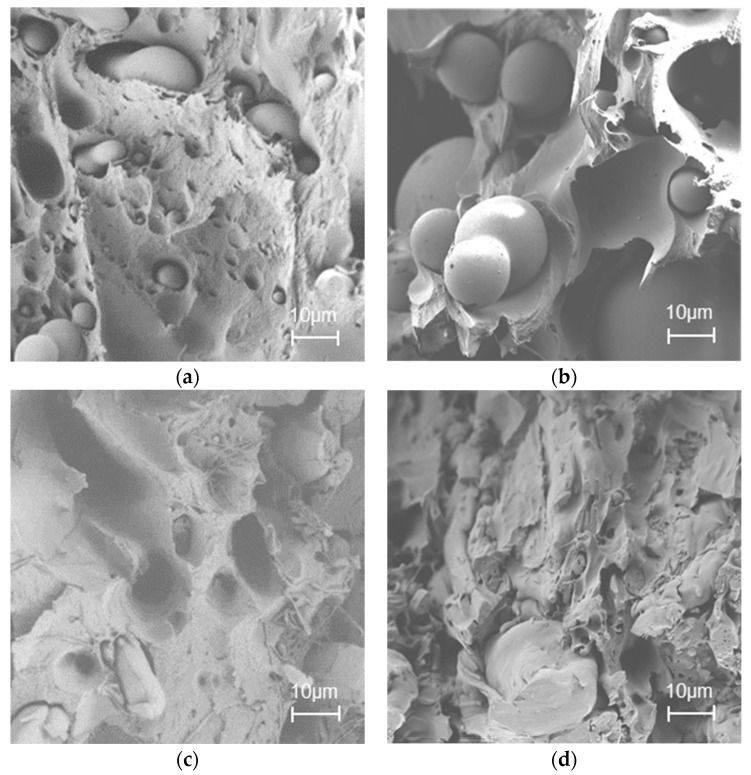
SEM images of blends at 1000× magnification (scale bar = 10 µm): (**a**) Cellulose C16/PLA 75%:25%; (**b**) cellulose C8/PLA 75%:25%; (**c**) cellulose C16/PLA 50%:50%; (**d**) cellulose C8/PLA 50%:50%.

According to the SEM results, in the cellulose C16/PLA and cellulose C8/PLA blends, spherical-shaped PLA droplets could be observed in the continuous cellulose ester matrix when the PLA ratios were 25 wt % and 50 wt % (Figure 4). Probably a longer compounding time or a higher compounding temperature would be needed to homogenize the PLA droplets into the cellulose ester matrix. However, it is known that high temperatures cause thermal degradation of cellulose. Therefore, a short extrusion time (1 min) was used to minimize this degradation. Another possible reason for droplets is that no additives were used during compounding, as was observed in an earlier reported study for nylon and LDPE blends. [47] When the PLA ratio was increased to 75 wt %, the matrix was more homogeneous. Then the PLA was uniformly dispersed in cellulose ester matrix and no clear PLA droplets were found (Figure S3, Supplementary Materials). Natural fibers are mainly used as reinforcements in polymeric composites, and so far, only limited literature is available regarding the use of cellulose esters in PLA or PE composites or blends. Lu et al. [21] have studied the interaction between cellulose fibers and the PLA matrix. They found that the interfacial adhesion between untreated cellulose fibers and the PLA matrix was very poor, but that adhesion was improved when cellulose fibers were modified. Our results support this observation, showing that the used esterified celluloses had moderate to good adhesion. 

The compatibility between bio-PE and cellulose ester matrices seems to be better than that between PLA and cellulose esters. However, some small unevenness was detected when 25 wt % bio-PE was added into cellulose esters (Figure S3, Supplementary Materials). Probably a longer compounding time or a higher compounding temperature would have been required to produce a uniform matrix. Pöllänen et al. [12] have studied the dispersion of cellulose fibers in HDPE matrix. They observed that cellulose fibers were dispersed rather well in the PE matrix, which is in line with our findings.

The densities of the compounded samples were analyzed, and detailed values are tabulated in Table 1. The densities of the blends correlated well with the material ratios used in the samples. The densities of cellulose C16, cellulose C8, and bio-PE were close to 1 g/mL, whereas the density of PLA was considerably higher (1.25 g/mL). By compounding the cellulose C8 and cellulose C16 with PLA, the density of PLA-based blends could be reduced by up to 15%, which is an advantage in many applications.

### 3.5. Mechanical Properties of Injection-Molded Cellulose Ester PE/PLA Blends 

**Figure 5:**
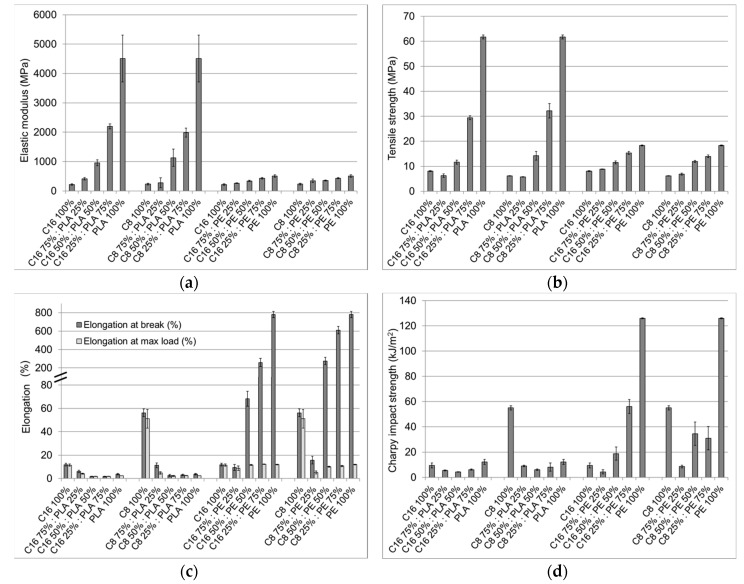
Mechanical properties of injection-molded blends: **(a)** Elastic modulus; **(b)** tensile strength; **(c)** elongation at break; **(d)** Charpy impact strength.

PLA had a very high elastic modulus (4500 MPa) compared to both pure cellulose C8 (233 MPa) and pure cellulose C16 (218 MPa) (Figure 5a). When 25 wt % cellulose ester was compounded with PLA, the elastic modulus values halved from 4500 MPa to 2200 MPa (C16) and 2000 MPa (C8). When the amount of cellulose esters was further increased, the elastic modulus of PLA blends decreased uniformly. Tensile strength values also showed the same trend (Figure 5b). This was the expected behavior because it is well known that the mechanical properties of blends depend on the properties of both components [48]. This trend is common for cellulose-based composites: Better interfacial adhesion results in higher strength. [49,50]. As we observed from the SEM analysis, PLA droplets formed in the continuous cellulose ester matrix, which decreased the mechanical properties of the blends. Matta et al. [51] reported the same effect with PCL/PLA composites. PCL spherulites in the PLA matrix acted as stress concentrators and thereby resulted in a decrease in tensile strength and tensile modulus.

The elastic modulus and tensile strength of bio-PE were also higher than those of cellulose C8 and cellulose C16. Similarly, mechanical values decreased evenly when bio-PE was compounded with cellulose esters (Figure 5a,b). Chollakup et al. [31] have studied the effects of unmodified fibers on HDPE mechanical properties. They found that elastic modulus increased from 130 MPa to 450 MPa when 20 wt % fiber was added to the HDPE matrix. The bio-PE used in our experiments had a much higher elastic value (512 MPa) than the HDPE used in Chollakup’s research, but in our study the addition of cellulose esters (25 wt %) decreased the elastic modulus by approximately 15%.

The elongation at break of PLA is rather low (3.7%) compared to cellulose C8 (56%) and cellulose C16 (12%) (Figure 5c). When PLA was compounded with cellulose esters, the elongation values already decreased close to the pure PLA elongation values with a 25 wt % PLA addition (25:75 ratio), and remained at the same level as other tested ratios.

Bio-PE had a very high elongation-at-break value (780%), whereas the elongation of cellulose-based esters was far from this value. However, the elongation of bio-PE at maximum load (12%) was at the same level as that of cellulose C16 (12%) and much lower than the elongation of cellulose C8 (51%). When cellulose C8 was compounded with bio-PE, the elongation at maximum load did not increase from the pure bio-PE values. The cellulose C8/bio-PE blend (75%:25%) had an exceptionally low elongation at maximum load value. This might be due to the poor interaction between bio-PE and cellulose esters, although it could not be clearly observed in the SEM analysis.

The impact strength of PLA was almost at the same level as cellulose C16, and was five-fold lower than that of cellulose C8 (Figure 5d). PLA is inherently a brittle polymer, and it seems that the brittleness was further increased by the addition of cellulose ester. When PLA was compounded with cellulose C16 or with cellulose C8 in different ratios, the impact strength decreased in all blends to below the value of pure PLA. This indicated the poor interaction between cellulose esters and PLA. Huda et al. [52] have studied the effect of unmodified cellulose fibers on PLA composites. They found that the impact strength of composites decreased when cellulose fibers were added and that the adhesion between the two phases was very poor. The same effect was observed for the cellulose ester/bio-PE blends. The impact strength of the compounded samples decreased with an increase in the cellulose ester concentration, because a more brittle cellulose ester filler had been added to the initially hard bio-PE matrix.

## 4. Conclusions

We showed that thermoplastic cellulose octanate (cellulose C8) and cellulose palmitate (cellulose C16) esters were completely processable without the addition of a plasticizer, which is very unusual in the case of cellulose esters. The elastic modulus and tensile strength of cellulose C16 and cellulose C8 were approximately the same, and no significant effect of side chain length on these properties was observed. However, cellulose C8 had five times higher elongation and impact strength than cellulose C16. The compatibility of these cellulose esters with PLA and bio-PE with different molar ratios was also tested. By compounding the cellulose C8 and cellulose C16 with PLA, the density of PLA-based blends could be reduced by up to 15%. In addition, cellulose C8 had significantly higher elongation and impact strength than PLA. The mechanical properties of cellulose esters and cellulose ester blends were below those of pure bio-PE. However, the results demonstrated that tested thermoplastic cellulose materials have good future potential in being used in injection molding applications.

## Figures and Tables

**Table 1:** Compounding temperatures and densities of compounded samples.

Material	Compounding Temperature (°C)	Density (g/mL)
C16 100%	130	0.99
C8 100%	130	1.05
PLA 100%	180	1.25
PE 100%	180	0.94
C16/C8 75%:25%	130	1.00
C16/C8 50%:50%	130	1.02
C16/C8 25%:75%	130	1.03
C16/PLA 75%:25%	180	1.05
C16/PLA 50%:50%	180	1.12
C16/PLA 25%:75%	180	1.18
C8/PLA 75%:25%	180	1.10
C8/PLA 50%:50%	180	1.15
C8/PLA 25%:75%	180	1.19
C16/PE 75%:25%	180	0.98
C16/PE 50%:50%	180	0.97
C16/PE 25%:75%	180	0.95
C8/PE 75%:25%	180	1.02
C8/PE 50%:50%	180	0.99
C8/PE 25%:75%	180	0.96

## Supplementary Materials

The following are available online at http://www.mdpi.com/1996-1944/11/12/2358/s1, Figure S1: Size exclusion chromatography (SEC) image of untreated and ozone-treated pulps, Figure S2: Solid state nuclear magnetic resonance (ssNMR) spectra of cellulose C8 and C16, Figure S3: SEM images of tested materials at 1000× magnification. Scale bar = 10 µm.
