# Supplementary material for: Injection Molding of Thermoplastic Cellulose Esters and Their Compatibility with Poly(Lactic Acid) and Polyethylene
Source: Materials (Basel). 2018 Nov 23;11(12):2358. doi: 10.3390/ma11122358 (PMC6316932; doi:10.3390/ma11122358)
Supplement: Supplementary file 1 [file materials-11-02358-s001.pdf]

materials

Supplementary: Injection Molding of Thermoplastic Cellulose Esters and Their Compatibility with POLY(lactic Acid) and Polyethylene

Pia Willberg-Keyriläinen, Hannes Orelma and Jarmo Ropponen

S1. Size exclusion chromatography (SEC)

The relative molar masses of the pulp samples were determined by size exclusion chromatography (SEC) measurements in 0.8% LiCl/DMAc eluent (0.36 mL/min, 80 °C) using MiniMix columns equipped with a Waters 2414 Refractive Index Detector (Waters, Milford, CT, USA). The relative molar mass distributions and average molar masses (Mn, Mw) were calculated against Pullulan Standards (6,100–1,600,000 g/mol). For SEC measurements, the pulp samples were dissolved in an 8% LiCl/DMAc, according to the solvent exchange method, with ethyl isocyanate derivatization to enhance the dissolution.

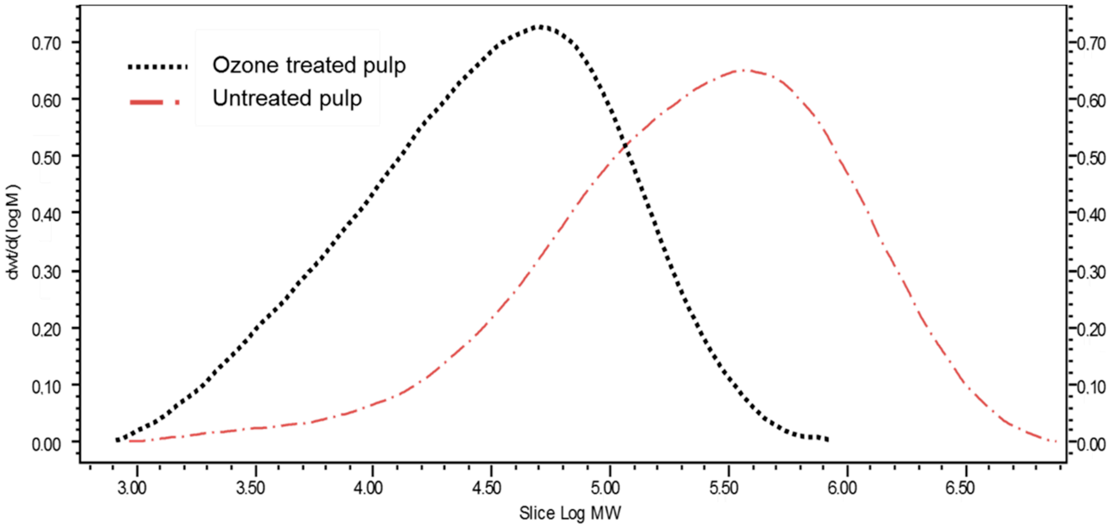

Figure S1. Size exclusion chromatography (SEC) image of untreated and ozone treated pulps.

S2. Solid state Nuclear magnetic resonance (ssNMR)

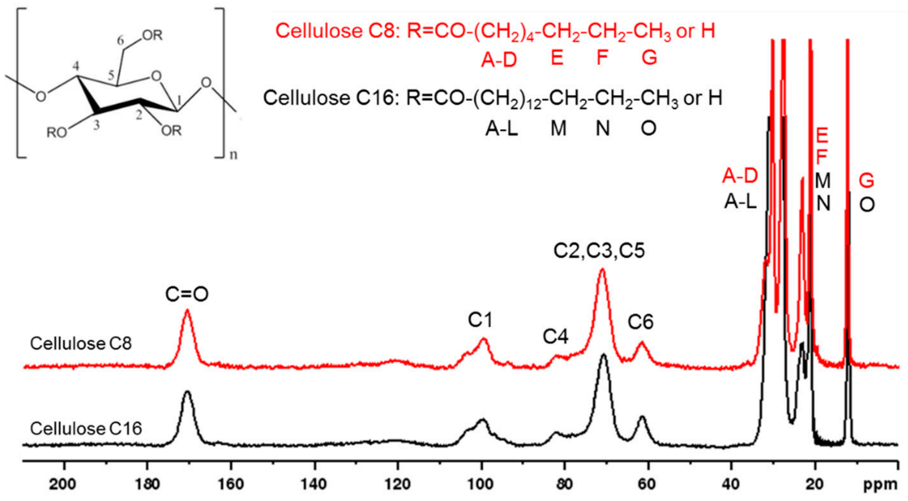

Figure S2. Solid state nuclear magnetic resonance (ssNMR) spectra of cellulose C8 and C16.

S3. Scanning Electron Microscopy (SEM)

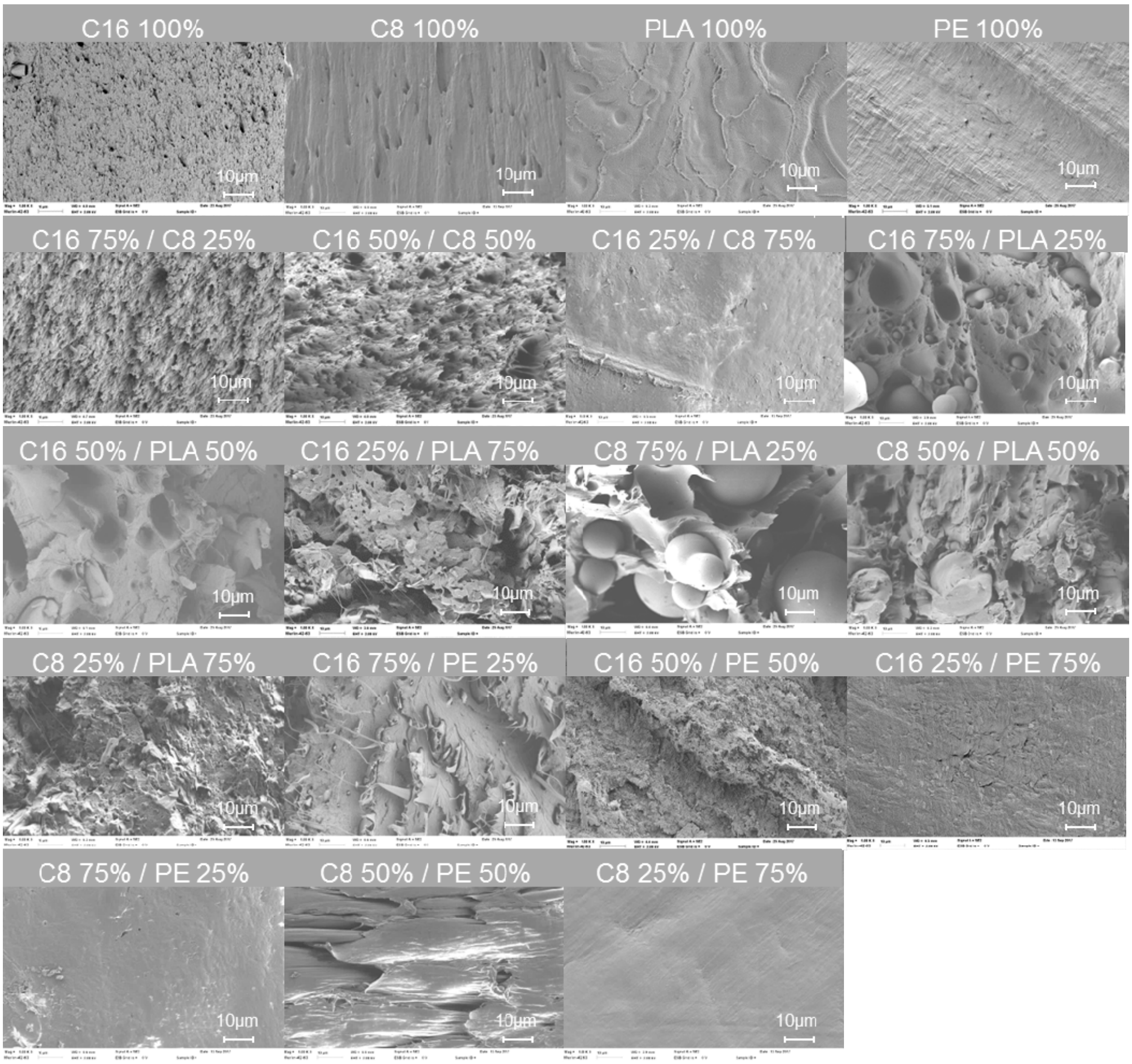

Figure S3. SEM images of tested materials at 1000× magnification. Scale bar = 10 μm.

© 2018 by the authors. Licensee MDPI, Basel, Switzerland. This article is an open access article distributed under the terms and conditions of the Creative Commons Attribution (CC BY) license (http://creativecommons.org/licenses/by/4.0/).
